# Supplementary figures and images for: N6-methyladenosine (m6A) modification in inflammation: a bibliometric analysis and literature review
Source: PeerJ. 2024 Dec 13;12:e18645. doi: 10.7717/peerj.18645 (PMC11648684; doi:10.7717/peerj.18645)

A

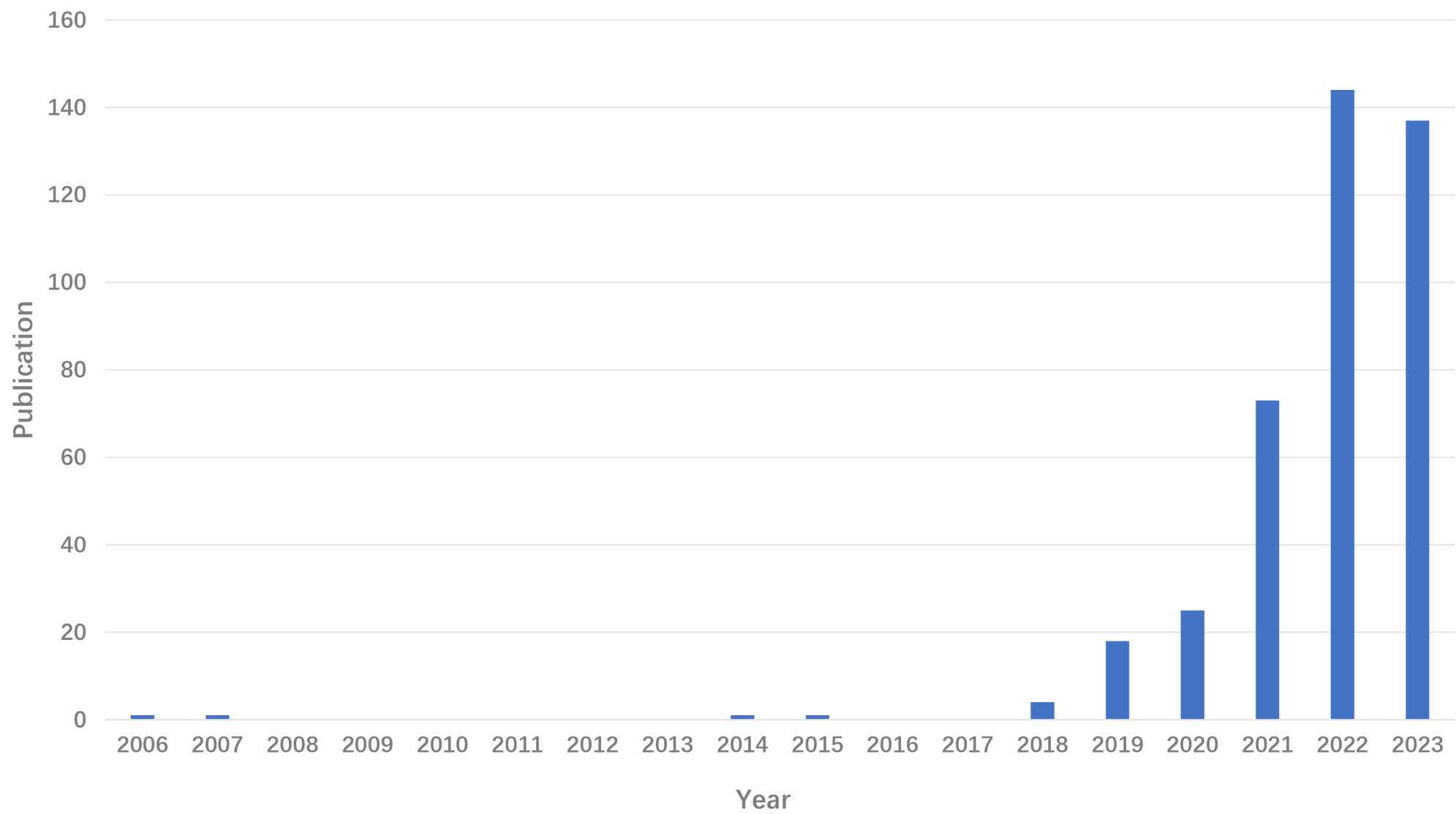

B

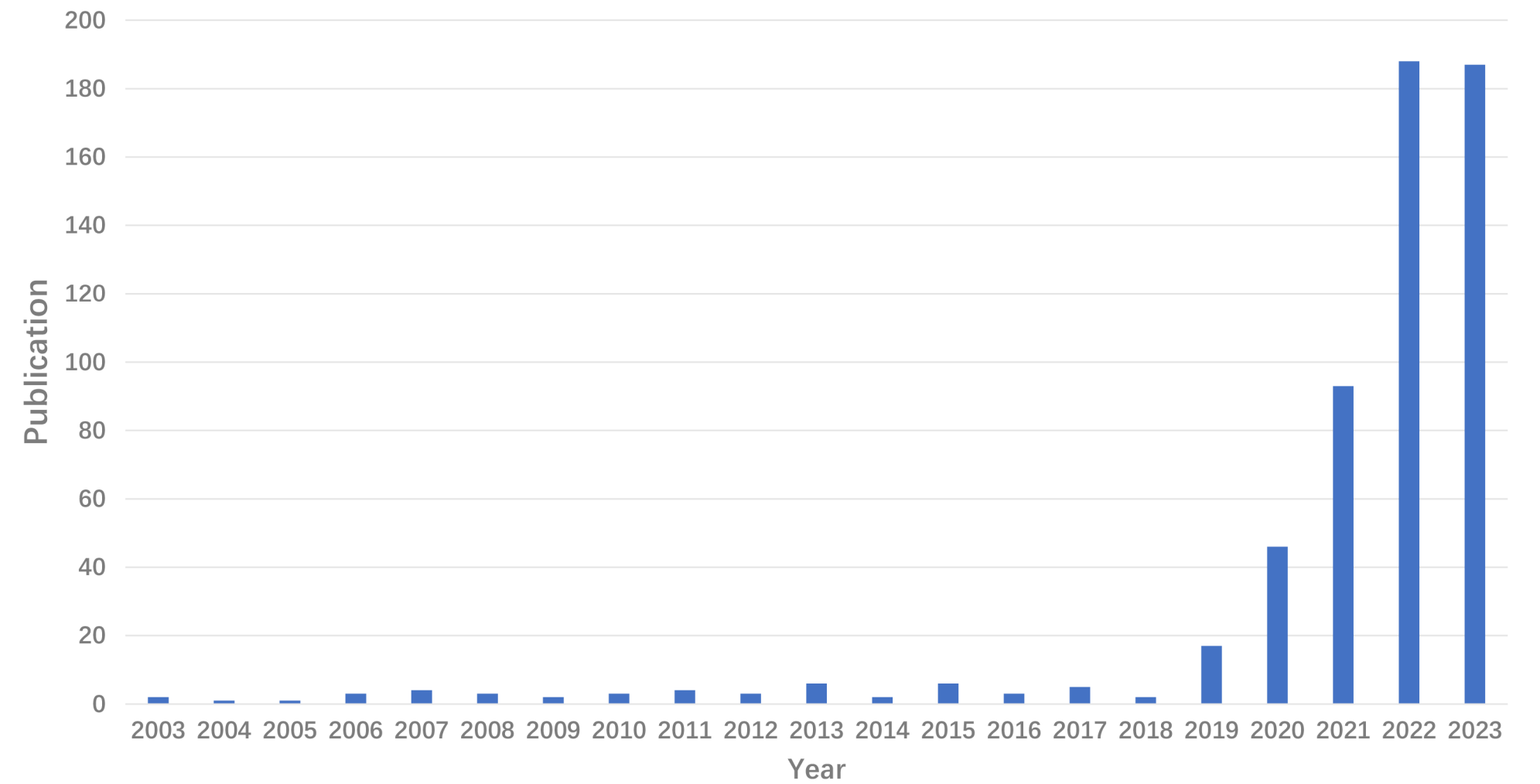

Supplement: Supplemental Information 1 [file peerj-12-18645-s001.pdf]

**A**

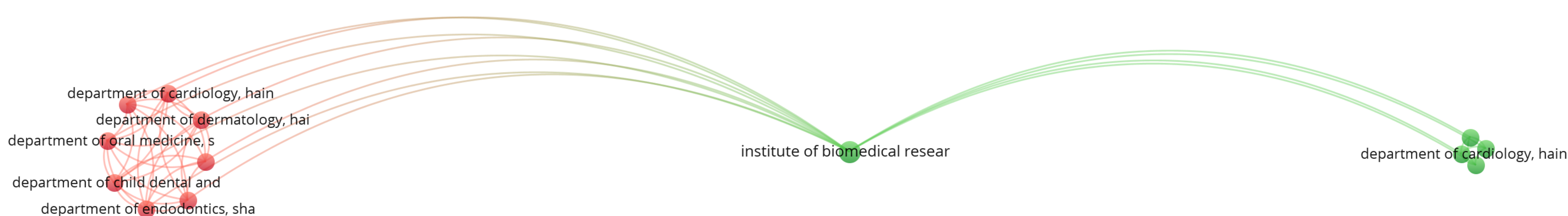 VOSviewer

# B

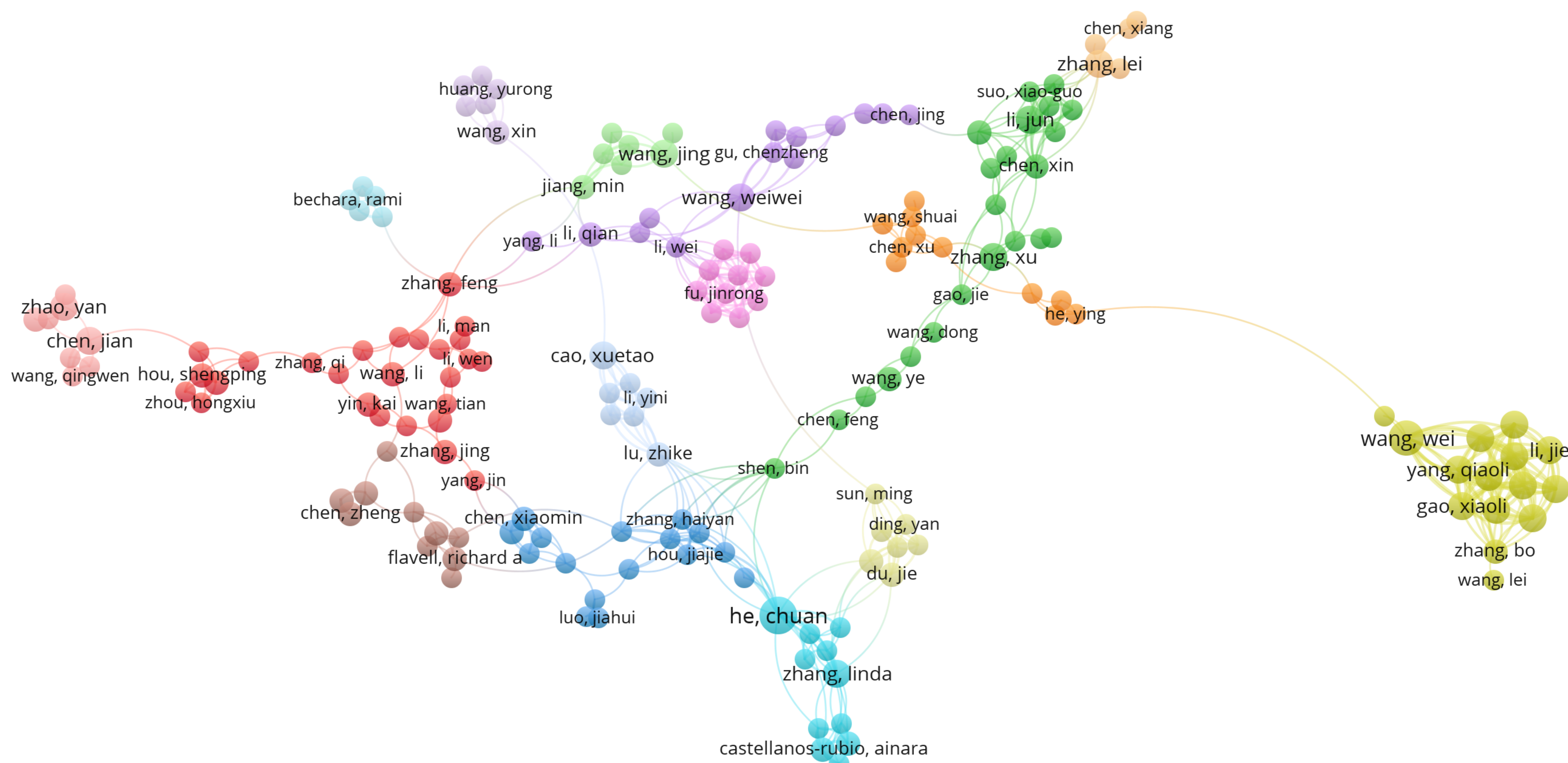 VOSviewer

C

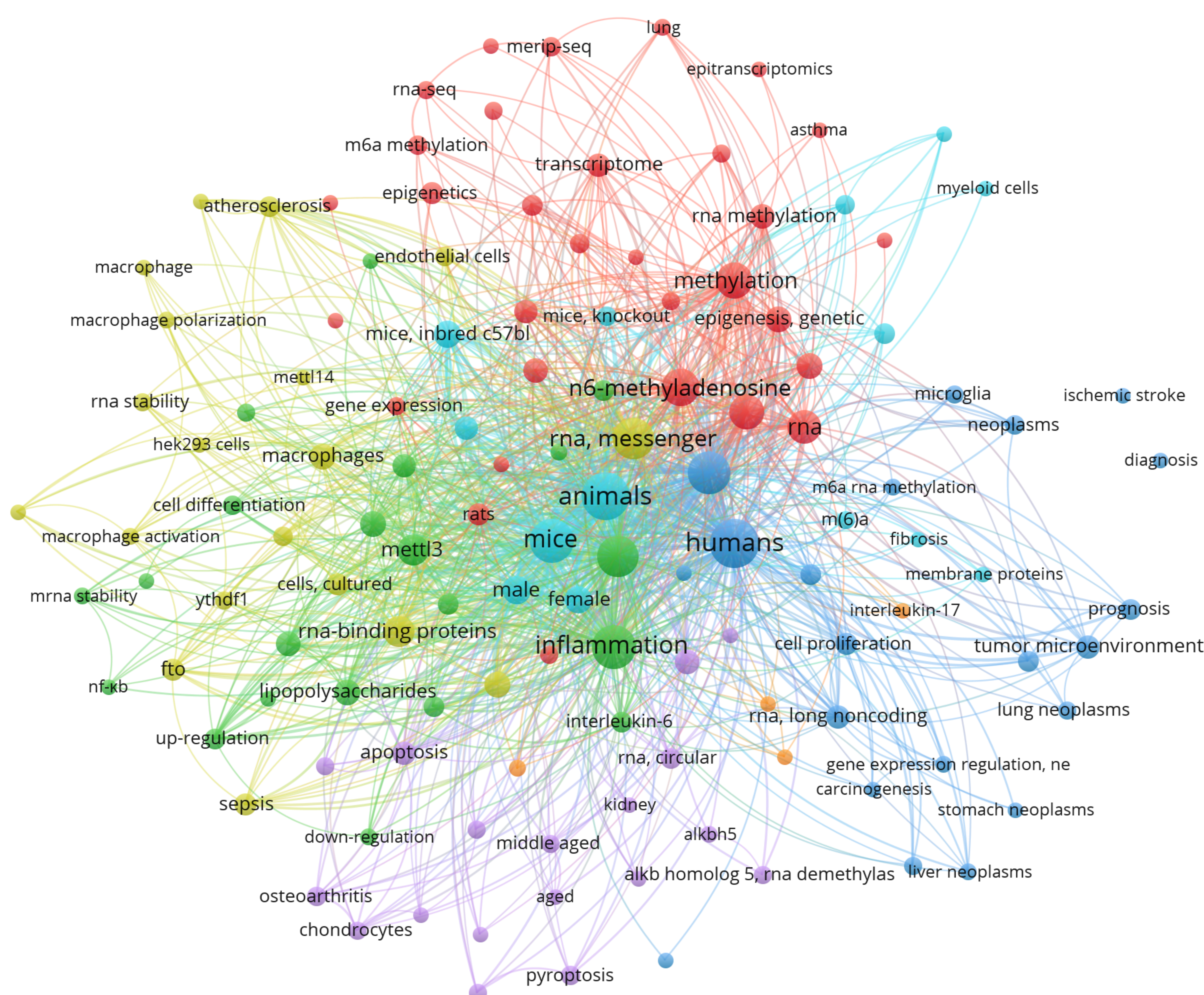 VOSviewer

Supplement: Supplemental Information 2 — Node and edge sizes are weighted by the number of published articles. The colors of nodes indicate different clusters. [file peerj-12-18645-s002.pdf]

A

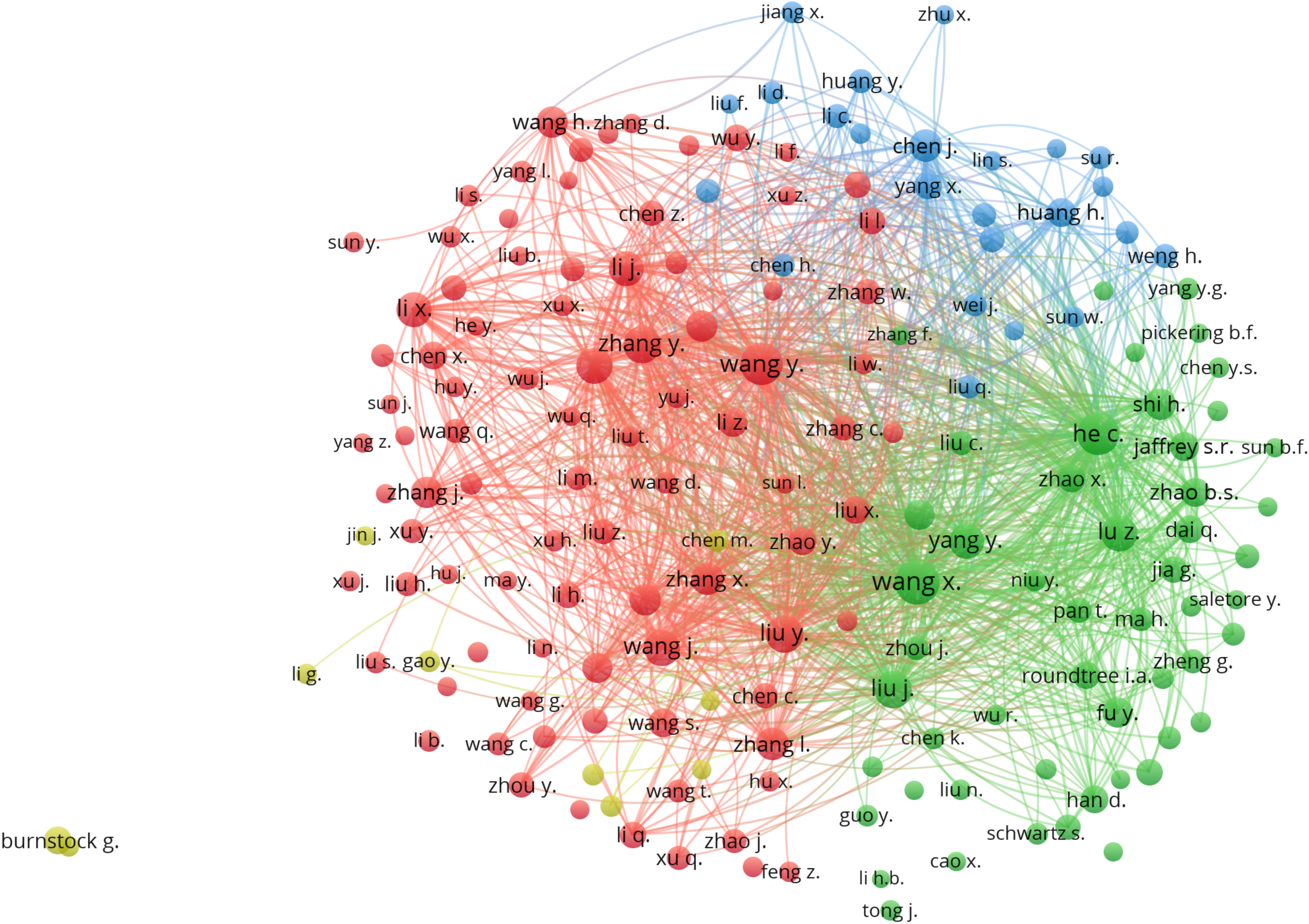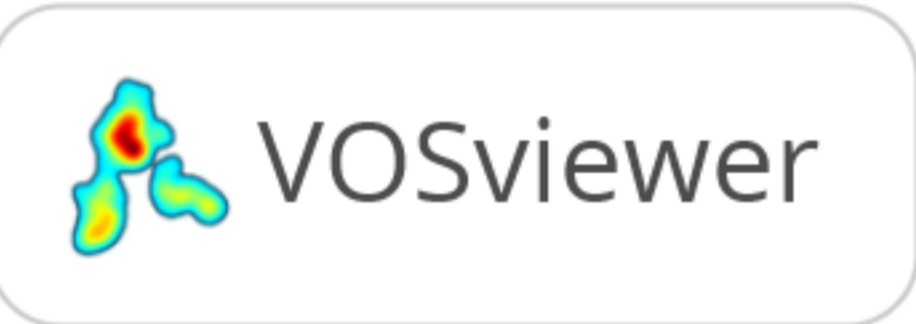

B

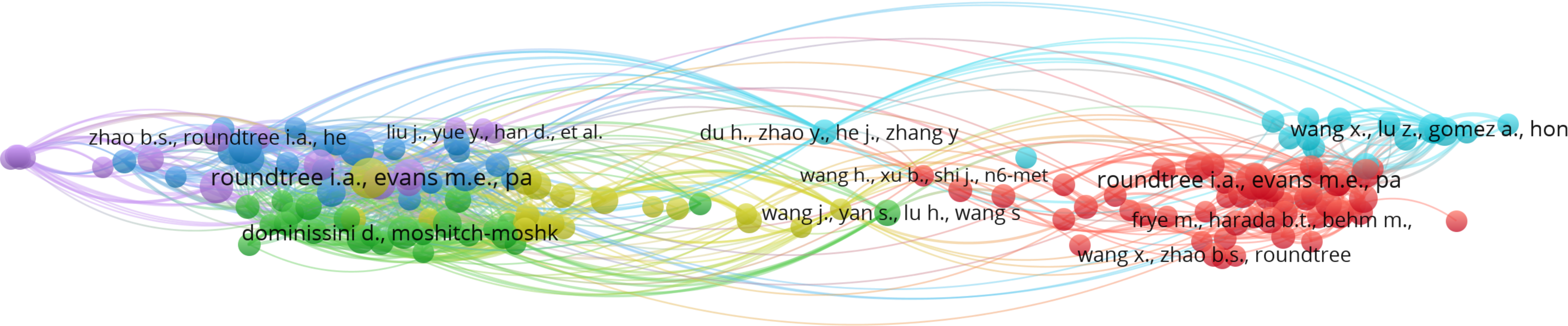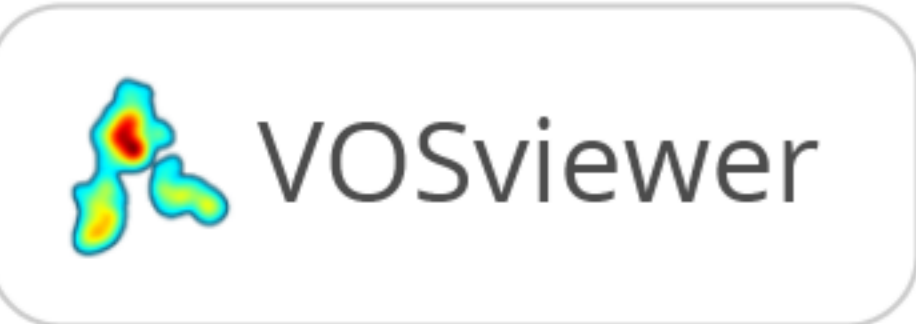

Supplement: Supplemental Information 4 — Node and edge sizes are weighted by the number of published articles. The colors of nodes indicate different clusters. [file peerj-12-18645-s004.pdf]
