# Supplementary material for: N6-methyladenosine (m6A) modification in inflammation: a bibliometric analysis and literature review
Source: PeerJ. 2024 Dec 13;12:e18645. doi: 10.7717/peerj.18645 (PMC11648684; doi:10.7717/peerj.18645)
Supplement: Supplemental Information 3 — The size of nodes is weighted by the number of published articles. [file peerj-12-18645-s003.pdf]

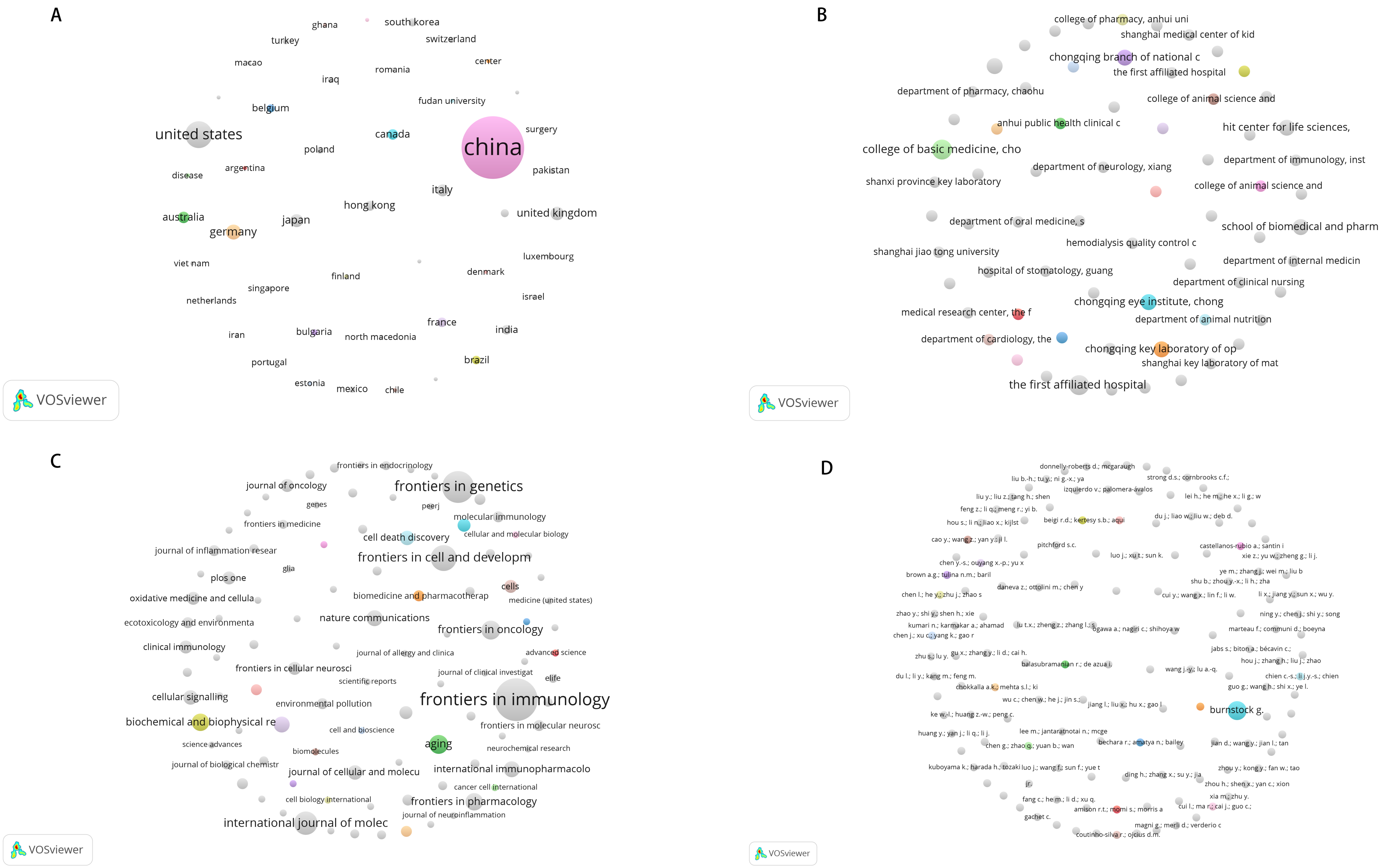

A
ghana
south korea
turkey
switzerland
macao
center
iraq
romania
fudan university
belgium
united states
canada
china
surgery
poland
pakistan
argentina
disease
italy
hong kong
united kingdom
australia
japan
germany
luxembourg
viet nam
finland
denmark
singapore
israel
netherlands
france
iran
bulgaria
north macedonia
india
portugal
brazil
estonia
mexico
chile
VOSviewer
B
college of pharmacy, anhui uni
shanghai medical center of kid
chongqing branch of national c
the first affiliated hospital
department of pharmacy, chaohu
college of animal science and
anhui public health clinical c
hit center for life sciences,
college of basic medicine, cho
department of immunology, inst
department of neurology, xiang
shanxi province key laboratory
college of animal science and
department of oral medicine, s
school of biomedical and pharm
hemodialysis quality control c
shanghai jiao tong university
department of internal medicin
hospital of stomatology, guang
department of clinical nursing
chongqing eye institute, chong
medical research center, the f
department of animal nutrition
department of cardiology, the
chongqing key laboratory of op
shanghai key laboratory of mat
the first affiliated hospital
VOSviewer
C
frontiers in endocrinology
journal of oncology
frontiers in genetics
genes
peerj
frontiers in medicine
molecular immunology
cellular and molecular biology
cell death discovery
journal of inflammation resear
frontiers in cell and developm
glia
plos one
cells
oxidative medicine and cellula
biomedicine and pharmacotherap
medicine (united states)
ecotoxicology and environmenta
nature communications
frontiers in oncology
clinical immunology
journal of allergy and clinica
advanced science
frontiers in cellular neurosci
journal of clinical investigat
scientific reports
elife
cellular signalling
environmental pollution
frontiers in immunology
biochemical and biophysical re
cell and bioscience
frontiers in molecular neurosc
science advances
aging
biomolecules
neurochemical research
journal of biological chemistr
journal of cellular and molecu
international immunopharmacolo
cancer cell international
cell biology international
frontiers in pharmacology
journal of neuroinflammation
international journal of molec
VOSviewer
D
donnelly-roberts d.; mcgaraugh
liu b.-h.; tu y.; ni g.-x.; ya
strong d.s.; cornbrooks c.f.;
izquierdo v.; palomera-ávalos
liu y.; liu z.; tang h.; shen
lei h.; he m.; he x.; li g.; w
feng z.; li q.; meng r.; yi b.
du j.; liao w.; liu w.; deb d.
hou s.; li n.; liao x.; kijlst
beigi r.d.; kertesy s.b.; aqui
cao y.; wang z.; yan y.; ji l.
pitchford s.c.
castellanos-rubio a.; santin i
luo j.; xu t.; sun k.
xie z.; yu w.; zheng g.; li j.
chen y.-s.; ouyang x.-p.; yu x
brown a.g.; tulina n.m.; baril
ye m.; zhang j.; wei m.; liu b
shu b.; zhou y.-x.; li h.; zha
daneva z.; ottolini m.; chen y
chen l.; he y.; zhu j.; zhao s
cui y.; wang x.; lin f.; li w.
li x.; jiang y.; sun x.; wu y.
zhao y.; shi y.; shen h.; xie
ning y.; chen j.; shi y.; song
kumari n.; karmakar a.; ahamad
lu t.x.; zheng z.; zhang l.; s
ogawa a.; nagiri c.; shihoya w
marteau f.; communi d.; boeyna
chen j.; xu c.; yang k.; gao r
jabs s.; biton a.; bécavin c.;
zhu s.; lu y.
gu x.; zhang y.; li d.; cai h.
hou j.; zhang h.; liu j.; zhao
balasubramanian r.; de azua i.
wang j.-y.; lu a.-q.
du l.; li y.; kang m.; feng m.
chien c.-s.; li j.y.-s.; chien
chokkalla a.k.; mehta s.l.; ki
guo g.; wang h.; shi x.; ye l.
wu c.; chen w.; he j.; jin s.;
jiang l.; liu x.; hu x.; gao l
burnstock g.
ke w.-l.; huang z.-w.; peng c.
lee m.; jantaratnotai n.; mcge
huang y.; yan j.; li q.; li j.
bechara r.; amatya n.; bailey
chen g.; zhao q.; yuan b.; wan
jian d.; wang y.; jian l.; tan
kuboyama k.; harada h.; tozaki
luo j.; wang f.; sun f.; yue t
zhou y.; kong y.; fan w.; tao
ding h.; zhang x.; su y.; jia
jr.
zhou h.; shen x.; yan c.; xion
xia m.; zhu y.
fang c.; he m.; li d.; xu q.
cui l.; ma r.; cai j.; guo c.;
amison r.t.; momi s.; morris a
gachet c.
magni g.; merli d.; verderio c
coutinho-silva r.; ojcius d.m.
VOSviewer
