# Supplementary material for: N6-methyladenosine (m6A) modification in inflammation: a bibliometric analysis and literature review
Source: PeerJ. 2024 Dec 13;12:e18645. doi: 10.7717/peerj.18645 (PMC11648684; doi:10.7717/peerj.18645)
Supplement: Supplemental Information 5 — The keyword co-occurrence network (A). The size of nodes and edges is weighted by the number of published articles. The colors of the nodes represent different clusters. The size of nodes and edges is weighted by the number of published articles. The colors of the nodes represent different clusters. Top 16 keywords with the strongest citation bursts (B). The years between “beginning” and “end” represent periods when keywords were more influential. Years in light green indicate that the keyword has not yet appeared, years in dark green indicate that the keyword has less influence, and years in red indicate that the keyword has more influence. The keywords Cluster Analysis (C). All of the keywords could be classified into seven categories, which were adenosine triphosphate, pulmonary fibrosis, nerve regeneration, tumor microenvironment, epitranscriptome, hypothermia, atherosclerosis. The timeline viewer of keywords.The timeline viewer of keywords (D). [file peerj-12-18645-s005.pdf]

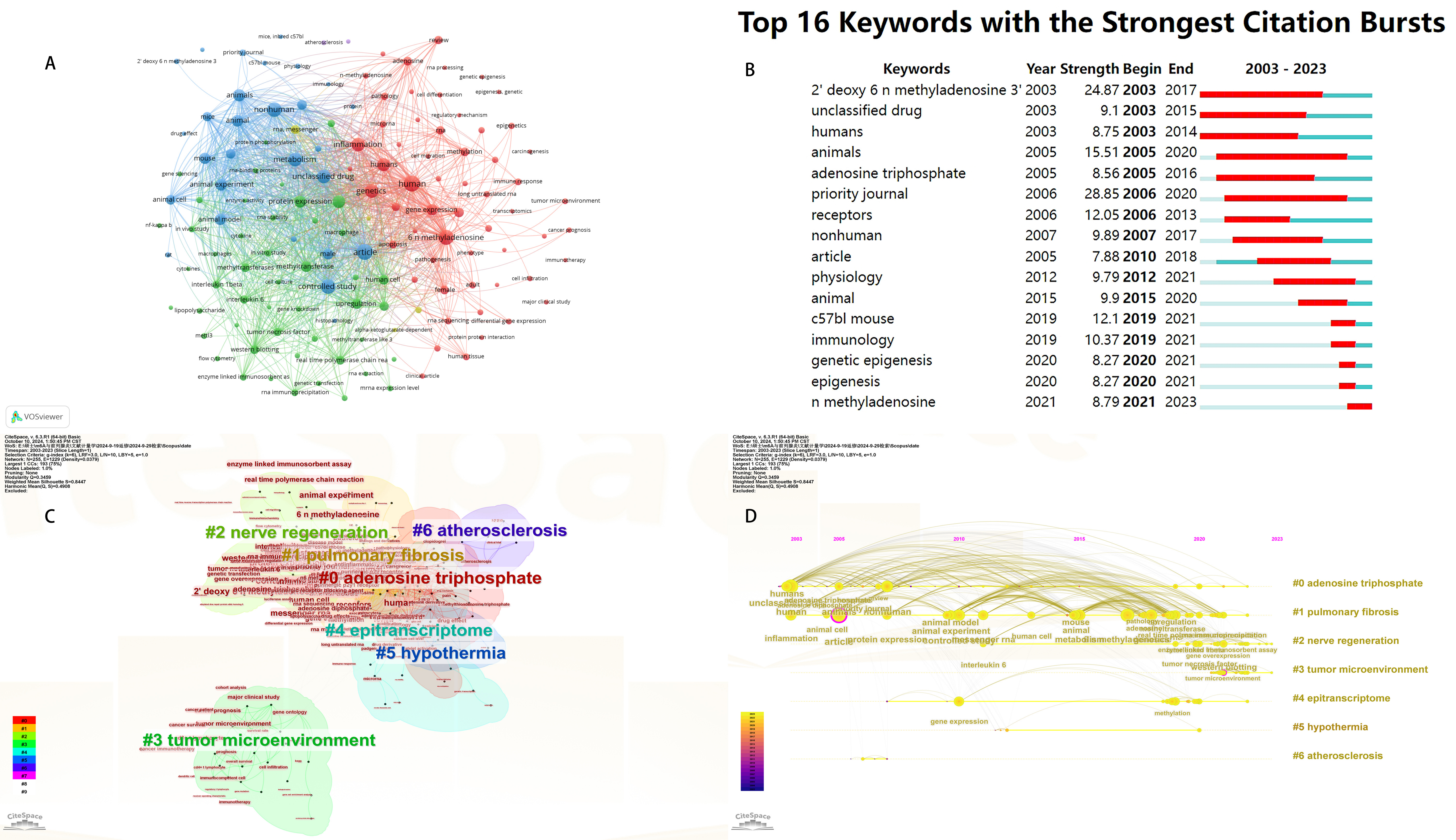
A
VOSviewer
mice, inbred c57bl
atherosclerosis
review
priority journal
2' deoxy 6 n methyladenosine 3
c57bl mouse
physiology
adenosine
rna processing
n-methyladenosine
genetic epigenesis
immunology
epigenesis, genetic
pathology
cell differentiation
animals
nonhuman
protein
regulatory mechanism
mice
animal
microrna
epigenetics
drug effect
rna, messenger
rna
protein phosphorylation
inflammation
mouse
metabolism
cell migration
methylation
carcinogenesis
humans
gene silencing
rna-binding proteins
unclassified drug
immune response
animal experiment
human
genetics
long untranslated rna
animal cell
enzyme activity
protein expression
tumor microenvironment
gene expression
transcriptomics
animal model
rna stability
nf-kappa b
in vivo study
macrophage
6 n methyladenosine
cancer prognosis
cytokine
apoptosis
rat
macrophages
in vitro study
male
article
phenotype
pathogenesis
immunotherapy
cytokines
methyltransferases
methyltransferase
human cell
cell infiltration
interleukin 1beta
cell culture
controlled study
adult
female
interleukin 6
upregulation
major clinical study
lipopolysaccharide
gene knockdown
histopathology
rna sequencing
differential gene expression
alpha-ketoglutarate-dependent
mettl3
tumor necrosis factor
methyltransferase like 3
protein protein interaction
western blotting
human tissue
flow cytometry
real time polymerase chain rea
rna extraction
clinical article
enzyme linked immunosorbent as
genetic transfection
rna immunoprecipitation
mrna expression level
Top 16 Keywords with the Strongest Citation Bursts
B
Keywords | Year | Strength | Begin | End | 2003 - 2023
2' deoxy 6 n methyladenosine 3' | 2003 | 24.87 | 2003 | 2017
unclassified drug | 2003 | 9.1 | 2003 | 2015
humans | 2003 | 8.75 | 2003 | 2014
animals | 2005 | 15.51 | 2005 | 2020
adenosine triphosphate | 2005 | 8.56 | 2005 | 2016
priority journal | 2006 | 28.85 | 2006 | 2020
receptors | 2006 | 12.05 | 2006 | 2013
nonhuman | 2007 | 9.89 | 2007 | 2017
article | 2005 | 7.88 | 2010 | 2018
physiology | 2012 | 9.79 | 2012 | 2021
animal | 2015 | 9.9 | 2015 | 2020
c57bl mouse | 2019 | 12.1 | 2019 | 2021
immunology | 2019 | 10.37 | 2019 | 2021
genetic epigenesis | 2020 | 8.27 | 2020 | 2021
epigenesis | 2020 | 8.27 | 2020 | 2021
n methyladenosine | 2021 | 8.79 | 2021 | 2023
C
CiteSpace, v. 6.3.R1 (64-bit) Basic
October 10, 2024, 1:50:45 PM CST
Timespan: 2003-2023 (Slice Length=1)
Selection Criteria: g-index (k=6), LRF=3.0, L/N=10, LBY=5, e=1.0
Network: N=255, E=1229 (Density=0.0379)
Largest 1 CCs: 193 (75%)
Nodes Labeled: 1.0%
Pruning: None
Modularity Q=0.3459
Weighted Mean Silhouette S=0.8447
Harmonic Mean(Q, S)=0.4908
Excluded:
enzyme linked immunosorbent assay
real time polymerase chain reaction
animal experiment
6 n methyladenosine
#2 nerve regeneration
#6 atherosclerosis
#1 pulmonary fibrosis
#0 adenosine triphosphate
#4 epitranscriptome
#5 hypothermia
#3 tumor microenvironment
major clinical study
cancer prognosis
gene ontology
cancer survival
tumor microenvironment
cancer immunotherapy
prognosis
overall survival
cell infiltration
immunocompetent cell
immunotherapy
CiteSpace
D
CiteSpace, v. 6.3.R1 (64-bit) Basic
October 10, 2024, 1:50:45 PM CST
Timespan: 2003-2023 (Slice Length=1)
Selection Criteria: g-index (k=6), LRF=3.0, L/N=10, LBY=5, e=1.0
Network: N=255, E=1229 (Density=0.0379)
Largest 1 CCs: 193 (75%)
Nodes Labeled: 1.0%
Pruning: None
Modularity Q=0.3459
Weighted Mean Silhouette S=0.8447
Harmonic Mean(Q, S)=0.4908
Excluded:
2003
2005
2010
2015
2020
2023
humans
human
inflammation
animal cell
article
animal model
animal experiment
protein expression
mouse
animal
human cell
interleukin 6
gene expression
methylation
tumor microenvironment
#0 adenosine triphosphate
#1 pulmonary fibrosis
#2 nerve regeneration
#3 tumor microenvironment
#4 epitranscriptome
#5 hypothermia
#6 atherosclerosis
CiteSpace
